# Clinical characteristics of hyperprogressive disease in NSCLC after treatment with immune checkpoint inhibitor: a systematic review and meta-analysis

**DOI:** 10.1186/s12885-020-07206-4

**Published:** 2020-07-29

**Authors:** Yan Chen, Junjie Hu, Fangfang Bu, Haiping Zhang, Ke Fei, Peng Zhang

**Affiliations:** 1 Department of Thoracic Surgery, Shanghai Pulmonary Hospital, Tongji University School of Medicine, No. 507 Zhengmin Road, Shanghai, 200433 China; 2 Department of Oncology, Shanghai Pulmonary Hospital, Tongji University School of Medicine, Shanghai, China

**Keywords:** Non-small cell lung cancer, Hyperprogressive disease, Immune checkpoint inhibitor, Immunotherapy, Meta-analysis

## Abstract

**Background:**

A number of studies have reported hyperprogressive disease (HPD) in non-small cell lung cancer (NSCLC) after treatment with immune checkpoint inhibitor (ICI). This study aimed to summarize the incidence and survival outcome of HPD in NSCLC and identify the clinicopathological features associated with HPD based on available eligible studies.

**Methods:**

Four databases (Medline/PubMed, Embase, Web of Science, and Cochrane Library) were searched for eligible studies on HPD published before January 23, 2020, to evaluate the incidence, outcome, and clinical features of HPD. Statistical analyses were performed using STATA 15.0. All meta-analyses were performed based on the random-effects model.

**Results:**

This study included 6 studies involving 1389 patients. The incidence of HPD ranged from 8.02 to 30.43%. Compared with patients with non-HPD, those with HPD were associated with worse overall survival. We identified that Eastern Cooperative Oncology Group > 1, Royal Marsden Hospital score ≥ 2, serum lactate dehydrogenase > upper limit of normal, the number of metastasis sites > 2, and liver metastasis were associated with the risk of HPD.

**Conclusions:**

This study summarized the clinical features of HPD in NSCLC patients. The meta-analysis showed that five pre-treatment clinicopathological features might be associated with HPD, which may help in selecting patients for ICIs.

## Background

Immune checkpoint inhibitor (ICIs) have shown sustained responses in different advanced-stage cancers, including non-small cell lung cancer (NSCLC) [1, 2]. Effects of ICIs on long-term survival of advanced NSCLC were tested in both first line and second line with randomized trials and showed a significant advantage over chemotherapy [3]. Theoretically, by interfering immunosuppressive programmed death-1/programmed death ligand-1 (PD-1/PD- L1) or cytotoxic T-lymphocyte antigen 4/B7 interactions, ICIs enhanced antitumor T cell activity and stimulated cancer-specific immune response thus improved prognosis. However, tumor immune microenvironment was complicated which might lead to an unpredictable response to ICIs. Increasing studies reported a new pattern of progression after initiation of ICI, which was termed as hyperprogressive disease (HPD) [4, 5]. Although rapid disease progression has also been described after other therapies [6, 7], several phase III studies showed a crossover between the immunotherapy and chemotherapy groups after initiating the therapies, suggesting that a higher proportion in the immunotherapy group had rapid disease progression in a short time after initiating ICIs [2, 8]. The definition of HPD varied in previous studies which were based on the different assessment approaches, such as tumor growth kinetics (TGK) and tumor growth rate (TGR), but the existence of this phenomenon had been proved. HPD has been reported across different tumor types, Inhwan Hwang suggested that the incidence and risk factors of HPD might differ according to cancer type [9]. It brings our minds to assess HPD in a specific cancer type. Currently, the clinical characteristics of HPD in NSCLC, such as the incidence, outcome and predictors of HPD are not well understood. A more profound understanding of HPD might help determine the position of ICIs in the management of NSCLC and identify patients who might progress after immunotherapy. Therefore, we performed this systematic review and meta-analysis to summarize the characteristics of HPD and evaluate the predictors of HPD in NSCLC.

## Methods

This study was conducted based on the Preferred Reporting Items for Systematic Reviews and Meta-Analysis statement [10].

### Literature search and study selection

Two independent authors (JH and YC) performed a systematic search of Medline/PubMed, Embase, Web of Science, and Cochrane Library databases for studies published before, January 23, 2020. The following key words were used for the search: hyperprogression or hyperprogressive disease. Language was restricted to English.

The studies were reviewed to evaluate the title, abstract, and full publication sequentially. The inclusion criteria were as follows: (1) clinical characteristics of HPD group and non-HPD group were described in NSCLC patients; (2) ICIs was used in the treatment; and (3) at least 30 patients were enrolled. Duplicate studies were excluded. Reviews, case reports, and studies not published as full studies, such as reference abstracts and letters to editors, were also excluded. The following results were compared to avoid the bias in this process, and all disagreements were resolved by discussion.

### Data extraction and quality assessment

Two authors (JH and YC) independently extracted data from the studies and assessed the risk of bias. All disagreements were resolved by consensus. The following data were obtained: the first author’s name, the year of publication, description of study population (number, age, gender, and geographic location), study design (prospective or retrospective), definition of HPD, clinicopathological features and survival outcome of HPD and non-HPD. Quality assessments were performed based on Newcastle–Ottawa Scale [11], which evaluated the study design based on 8 questions about the population selection, comparability, and exposure.

### Definitions and statistical analysis

This meta-analysis was conducted to report clinicopathological features of HPD in NSCLC. As no standard criteria exist to define HPD, the criteria reported in each included study were accepted in this study (Table 1). TGR was calculated according to Champiat [4] as the log-scale calibrated change in the sum of the volumes of the target lesions according to RECIST 1.1 criteria [12] per month. The TGRpost/TGRpre was considered to be the ratio of the TGR between the baseline and the first imaging after initiation of ICIs to the TGR between the pre-baseline and baseline. The definition of TGK was different in two studies. In the study of Kim CG, TGK was defined as the difference in the sum of the largest diameters of the target lesions according to RECIST 1.1 per month [13]. In the study of Kim Y, TGK was defined as the difference of total tumor volume per month [14]. The TGKpost/TGKpre was calculated as the ratio of the TGK between the baseline and the first imaging after ICIs treatment to the TGK between the pre-baseline and baseline.

**Table 1:** Definition of hyperprogressive disease in each included study

Study	Definition of HPD
Ferrara R [7]	PD at first evaluation and (TGRpost-TGRpre)/ TGRpre^a^ > 50%
Lo Russo G [15]	Fulfilling at least 3 of the following 5 criteria: (1) Time to treatment failure < 2 months; (2) > 50% increase in the sum of target lesions major diameters between baseline and first radiologic evaluation; (3) appearance of at least two new lesions in an organ already involved between baseline and first radiologic evaluation; (4) spread of the disease to a new organ between baseline and first radiologic evaluation; 5) ECOG ≥2 during the first 2 months of treatment
Tunali I [16]	PD at first evaluation, TGRpost/TGRpre^a^ ≥ 2 and time to treatment failure < 2 months
Kim CG [13]	PD at first evaluation, TGRpost/TGRpre^a^ ≥ 2 and TGKpost/TGKpre^b^ ≥ 2
Kim Y [14]	PD at first evaluation, TGKpost/TGKpre^c^ ≥ 2, time to treatment failure < 2 months and > 50% increase of total tumor volume compared with baseline volume
Castello A [17]	The same criteria proposed by Lo Russo G

*ECOG* Eastern Cooperative Oncology Group, *HPD* hyperprogressive disease, *PD* progressive disease at the first response evaluation after treatment, *TGK* tumor growth kinetics, *TGR* tumor growth rate

^a^TGR was calculated according to Champiat et al [4] as the log-scale calibrated change in the sum of the volumes of the target lesions according to RECIST 1.1 criteria per month

^b^TGK was defined as the difference in the sum of the largest diameters of the target lesions according to RECIST 1.1 per month

^c^TGK was defined as the difference of total tumor volume per month

The pooled odds ratio (OR) with 95% confidence interval (CI) were calculated to evaluate the association between clinicopathological features and risk of HPD. A random-effects (DerSimonian–Laird method) model was used. The impact of statistical heterogeneity was assessed using the χ^2^-based Q test and I^2^ test, with heterogeneity *P* < 0.1 or I^2^ > 50% considered to indicate a statistically significant difference. Publication bias was evaluated with Egger’s test. A *P* value < 0.05 was considered statistically significant. The Stata 15.0 software (Stata Corporation, TX, USA) was used to perform all the tests.

## Results

**Fig. 1:**
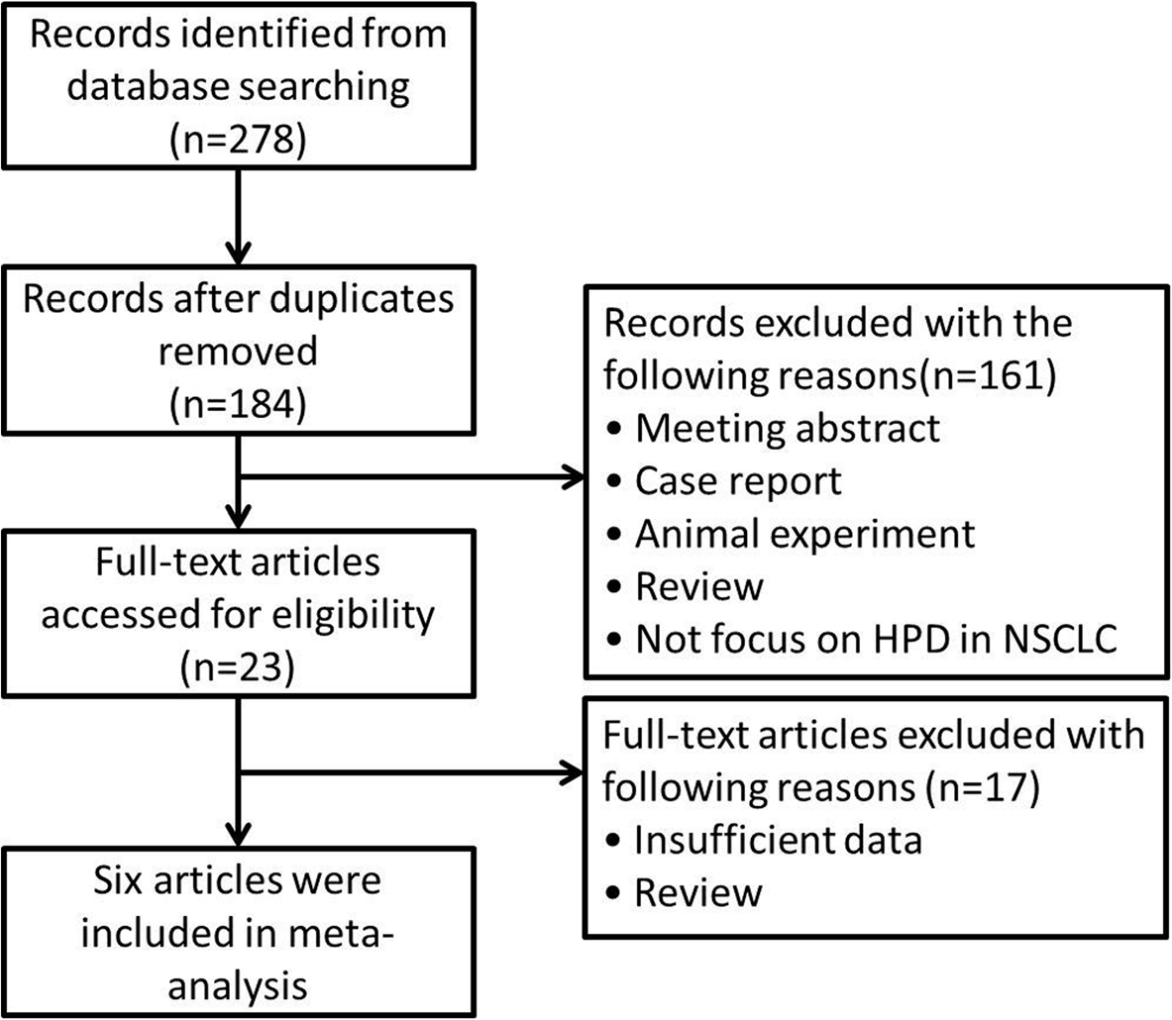
Flowchart for study selection. HPD, hyperprogressive disease

Figure 1 showed that the literature search identified 278 studies from the 4 databases. After screening the titles and abstracts, 161 studies were excluded because they were review articles, case reports, letters, conference abstracts, or not related to HPD. Next, 23 studies were identified for further review in full text, of which 17 were eliminated because no sufficient data was reported about HPD and non-HPD group. Finally, six studies were included in the meta-analysis [7, 13–17]. The quality scores of the all 6 identified studies were 6.

The definition of HPD varied in the included studies. Lo Russo and Castello adopted criteria combined clinical and radiologic parameters [15, 17]. Other studies evaluated the evolution of tumor volume or the sum of the largest diameters based on three sequential imaging (before, at the start, and during ICI). Ferrara adopted 50% as the threshold of the difference between the TGR at pre-treatment and post –treatment [7]. Kim CG defined HPD based on a 2-fold increase in TGR and TGK according to RECIST 1.1 criteria which showed a high concordance rate [13]. Kim Y and his colleagues evaluated HPD based on the difference in the total volume of tumor per unit of time [14].

Table 2 showed the characteristics of the studies included in this systematic review. The 6 retrospective studies represented 1349 patients from the United States, France, Italy and Korea. All eligible studies were retrospective. Except for the study of Ferrara, which had a control cohort treated with chemotherapy, all other studies were single-arm studies [7]. Kim Y classified patients having PD by RECIST 1.1 as HPD and non-HPD groups, other studies classified all NSCLC patients treated with ICIs as HPD and non-HPD groups [14]. The number of patients in each study ranged from 46 to 406. The incidence of HPD in NSCLC ranged from 8.02 to 30.43%. Lo Russo and Castello compared the survival outcome of HPD and non-HPD patients, other studies compared prognosis of HPD and PD without HPD patients [15, 17]. HPD patients were associated with significantly worse OS in all included studies. Further meta-analysis of incidence and OS of HPD were not performed for existence of heterogeneity.

**Table 2:** Characteristics of eligible studies

Study	Year	Country	Study design	Patient	HPD	Incidence of HPD	Overall survival	NOS
Ferrara R [7]	2018	France	Retrospective	406	56	13.79%	HPD vs. PD without HPD (HR 2.18, 95% CI (1.29–3.69), *p* = 0.03)	6
Lo Russo G [15]	2018	Italy	Retrospective	152	39	25.66%	HPD vs. non-HPD (4.4 vs. 17.7 months)	6
Tunali I [16]	2019	USA	Retrospective	187	15	8.02%	HPD vs. PD without HPD (3.2 vs. 8.4 months, *p* < 0.001)	6
Kim CG [13]	2019	Korea	Retrospective	263	54	20.53%	HPD vs. PD without HPD (HR 5.71, 95% CI 3.14–8.23, *p* < 0.05)	6
Kim Y [14]	2019	Korea	Retrospective	335	48	14.33%	HPD vs. PD without HPD (HR 1.9, 95% CI 1.2–3.0, *p* = 0.006)	6
Castello A [17]	2019	Italy	Retrospective	46	14	30.43%	HPD vs. non-HPD (4 vs. 15 months, *p* = 0.003)	6

*HPD* hyperprogressive disease, *NOS* Newcastle–Ottawa Scale, *PD* progressive disease at the first response evaluation after treatment, *USA* the United States

**Fig. 2:**
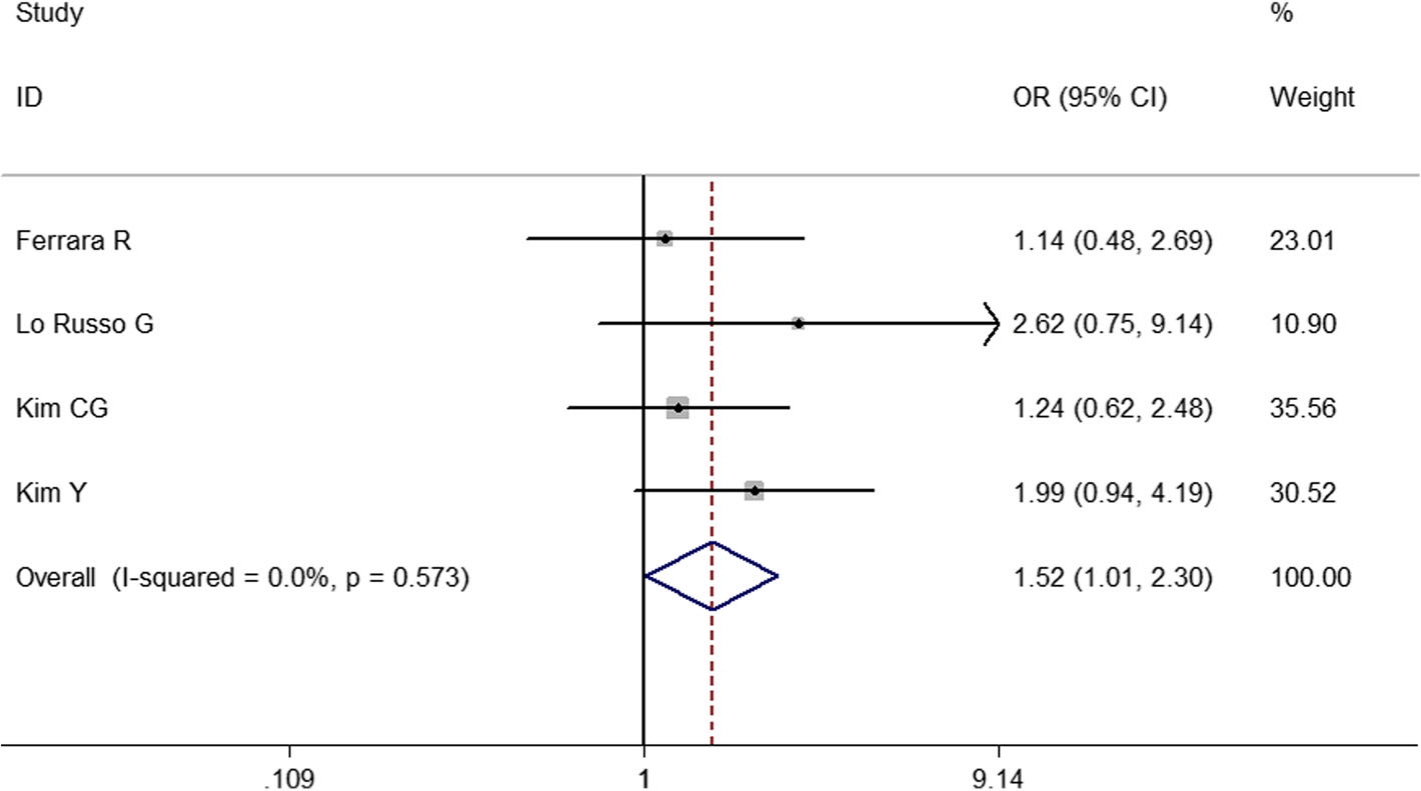
Forest plot of the association between Eastern Cooperative Oncology Group and hyperprogressive disease. OR, odd ratio; CI, confidence interval

**Fig. 3:**
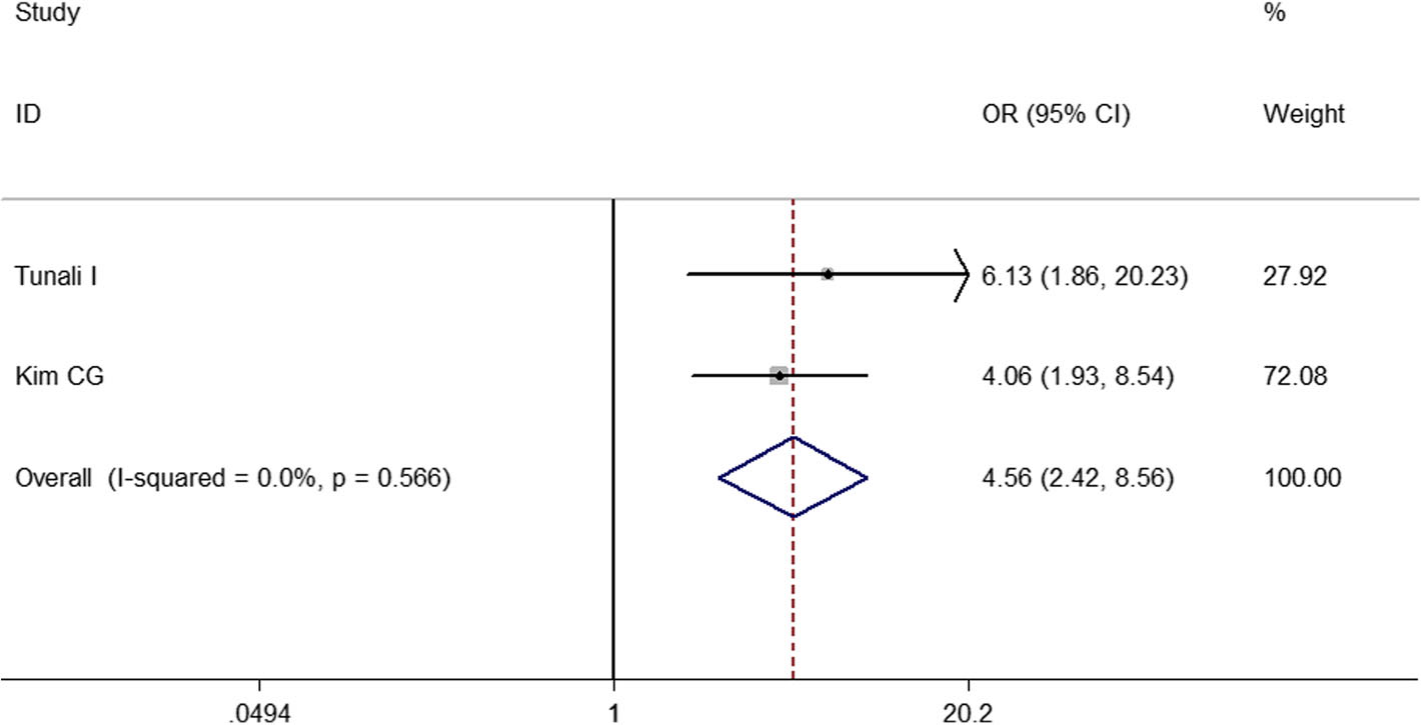
Forest plot of the association between Royal Marsden Hospital score and hyperprogressive disease. OR, odd ratio; CI, confidence interval

**Fig. 4:**
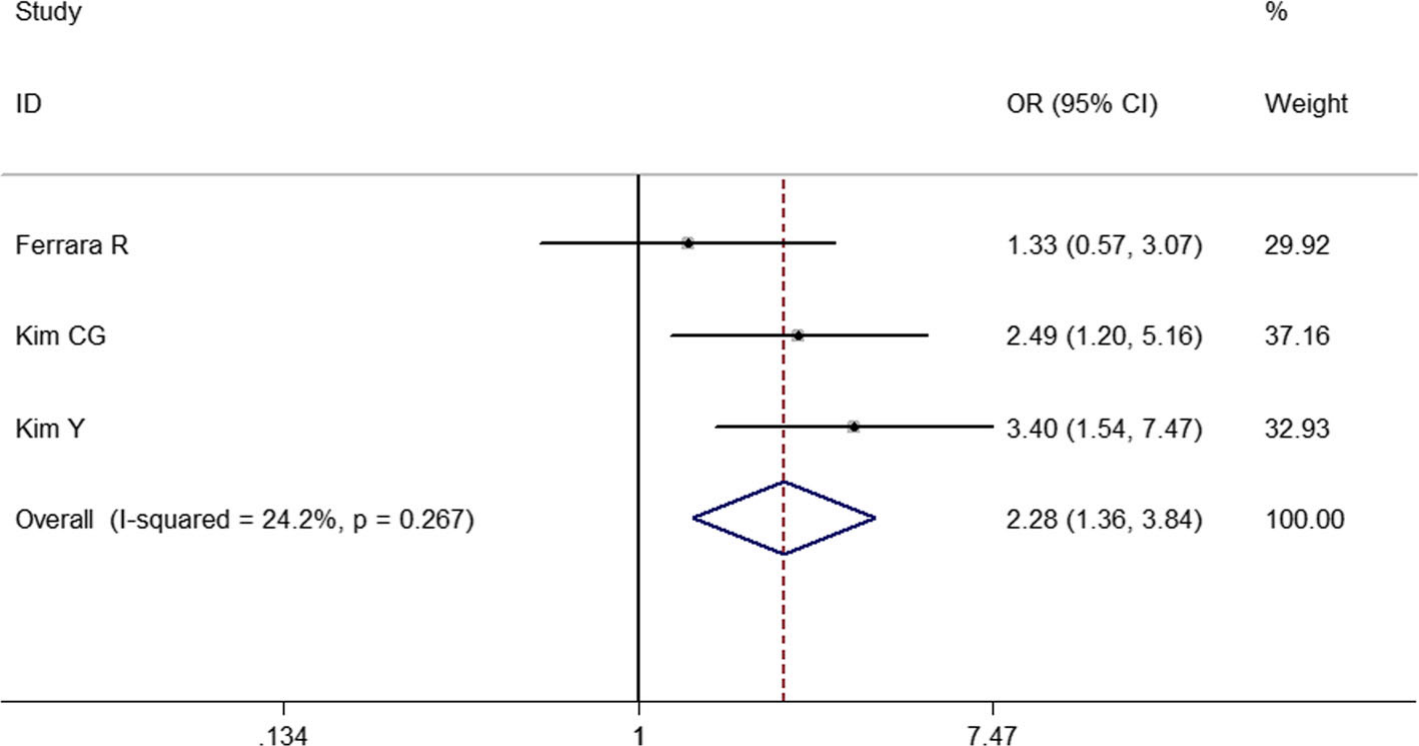
Forest plot of the association between serum lactate dehydrogenase and hyperprogressive disease. OR, odd ratio; CI, confidence interval

**Fig. 5:**
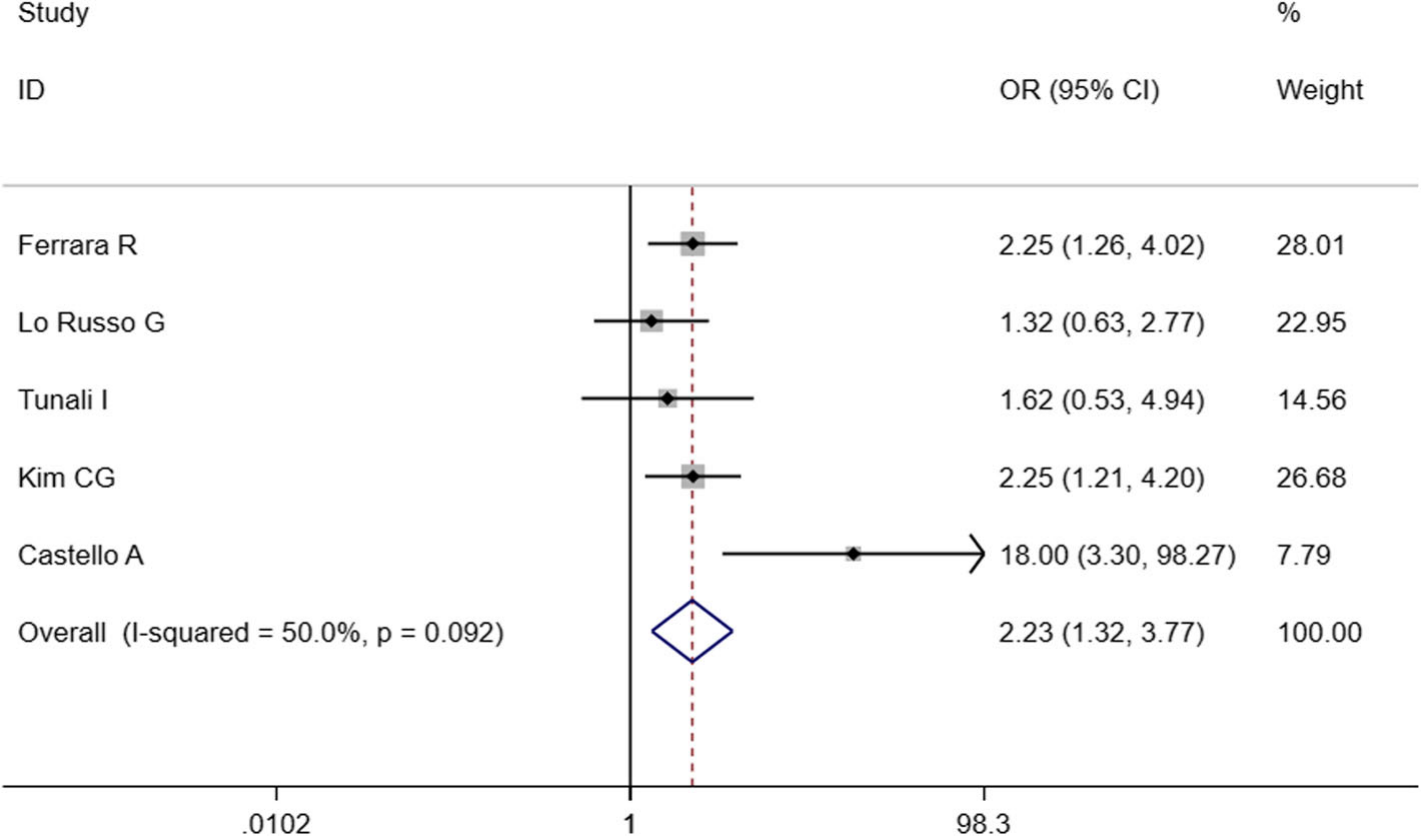
Forest plot of the association between the number of metastasis sites and hyperprogressive disease. OR, odd ratio; CI, confidence interval

**Fig. 6:**
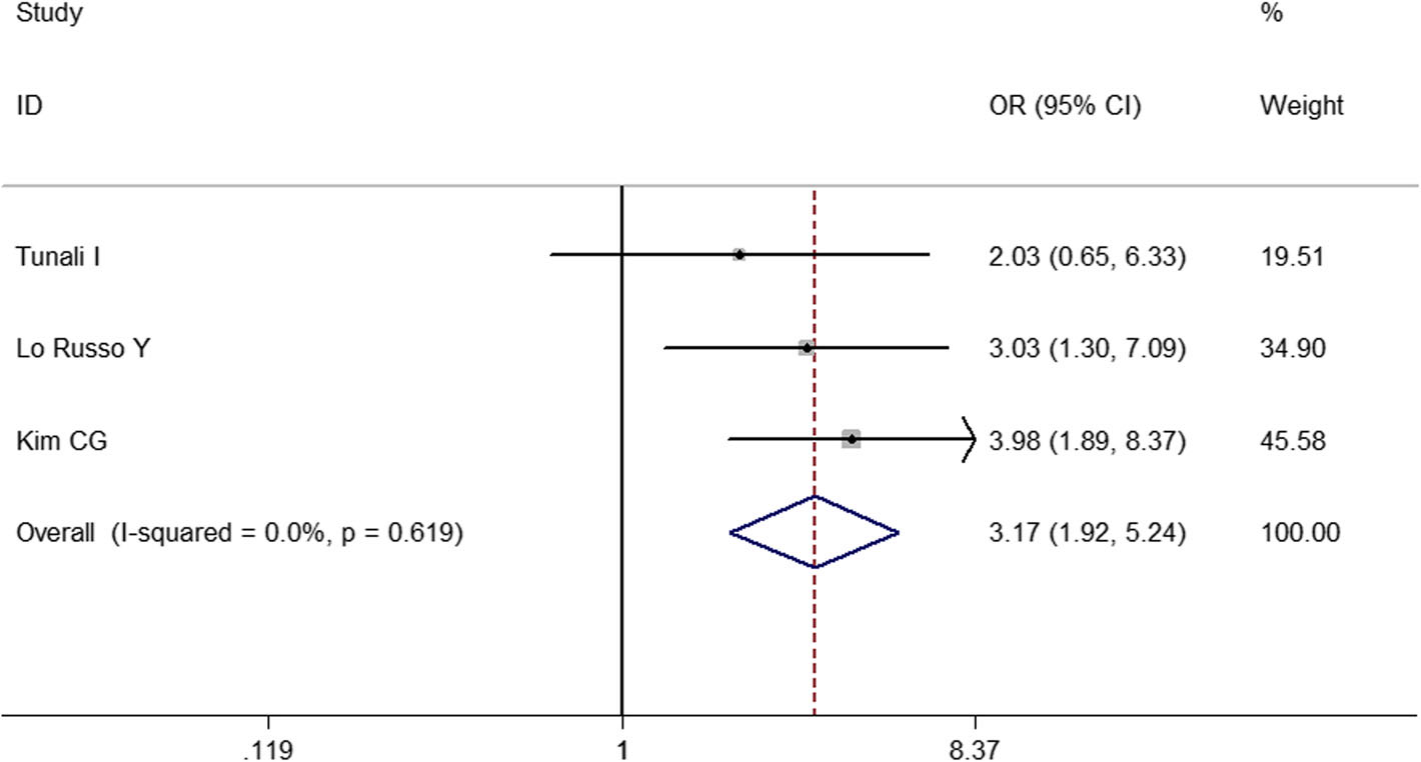
Forest plot of the association between liver metastasis and hyperprogressive disease. OR, odd ratio; CI, confidence interval

To identify predictive factors of HPD, we performed meta-analysis on 14 clinicopathological features (Table 3). We identified 5 different factors significantly associated with the risk of HPD (Figs. 2, 3, 4, 5 and 6): Eastern Cooperative Oncology Group (ECOG) > 1 (OR = 1.524; 95% CI, 1.009–2.301; *P* = 0.045), Royal Marsden Hospital (RMH) score ≥ 2 (OR = 4.556; 95% CI, 2.424–8.561; *P* < 0.001), serum lactate dehydrogenase > upper limit of normal (OR = 2.285; 95% CI, 1.360–3.839; *P* = 0.002), the number of metastasis sites > 2 (OR = 2.231; 95% CI, 1.321–3.767; *P* = 0.003), and liver metastasis (OR = 3.173; 95% CI, 1.920–5.244; *P* < 0.001). Serum lactate dehydrogenase more than upper normal limit showed mild heterogeneity (*I*^*2*^ = 24.3%), more than 2 metastasis sites showed middle heterogeneity (*I*^*2*^ = 50.0%), but the effect direction of the individual studies was consistent. The other 4 factors that correlated with HPD didn’t show any heterogeneity. For only 2 studies included in the association of RMH, egger P was not available. Publication bias evaluation for the other 5 factors revealed that there was no significant publication bias. No significant correlation of HPD was found with age > 65 years, gender, smoking history, neutrophil-to-lymphocyte ratio, PD1/PD-L1, PD-L1 status, monotherapy/combination, number of previous treatment lines, pathological pattern in NSCLC, EGFR mutation, KRAS mutation, or ALK rearrangement in NSCLC.

**Table 3:** Associations between hyperprogressive disease and clinicopathological features

Clinical parameter	*N*, studies	*N*, patients	Overall OR	95% CI	*I*^2^ (%)	Significance (*P*)	Egger *P*
Age ≥ 65 years vs < 65 years	2	593	0.818	0.490–1.364	0	0.441	NA
Male vs female	5	783	0.812	0.556–1.185	4.3	0.280	0.743
Ever smoker vs nerver smoker	5	774	0.955	0.641–1.423	0.5	0.823	0.106
**ECOG > 1 vs ≤ 1**	**4**	**965**	**1.524**	**1.009–2.301**	**0**	**0.045**	**0.471**
**RMH ≥ 2 vs < 2**	**2**	**332**	**4.556**	**2.424–8.561**	**0**	**< 0.001**	**NA**
Neutrophil-to-lymphocyte ratio ≤ 3 vs > 3	3	680	0.595	0.265–1.334	73.5	0.208	0.747
**Serum lactate dehydrogenase > upper normal limit**	**3**	**493**	**2.285**	**1.360–3.839**	**24.2**	**0.002**	**0.606**
**No. of metastasis > 2 vs ≤ 2**	**5**	**1054**	**2.231**	**1.321–3.767**	**50**	**0.003**	**0.339**
**Liver metastasis**	**3**	**602**	**3.173**	**1.920–5.244**	**0**	**< 0.001**	**0.109**
PD-1 vs PD-L1	4	930	1.497	0.875–2.561	0	0.141	0.946
PD-L1 positive	5	546	0.776	0.499–1.205	0	0.259	0.460
Monotherapy vs combination	2	557	0.511	0.033–7.898	83.3	0.631	NA
Previous treatment lines > 2	4	856	0.741	0.394–1.393	70.5	0.352	0.923
Squamous	5	1143	0.832	0.587–1.179	0	0.301	0.828
EGFR mutation	5	928	0.956	0.537–1.705	0	0.880	0.148
KRAS mutation	3	487	0.992	0.535–1.840	0	0.980	0.502
ALK rearrangement	3	660	2.860	0.652–12.547	0	0.164	0.151

**Abbreviations:***CI* Confidence interval, *ECOG* Eastern Cooperative Oncology Group, *HPD* hyperprogressive disease, *NSCLC* non-small-cell lung cancer, *OR* odds ratio, *PD-1* programmed death-1, *PD-L1* programmed death ligand-1, *RMH* Royal Marsden Hospital

## Discussion

ICIs have shown promising effects in treating advanced NSCLC. However, increasing evidence reported the association of rapid progression or HPD with ICIs. Our study summarized current data about the incidence, outcome, and clinicopathological features of HPD. In the present studies, the incidence of HPD ranged from 8.02 to 25.66% in NSCLC. Among the included studies, only one study contained a chemotherapy cohort [7], which reported a 5.1% (3/59) incidence of HPD in patients with NSCLC treated with single-agent chemotherapy. The result of the present study was consistent with the findings of previous phase III trials that OS curves crossed between 3 and 6 months [2, 8], suggesting that a higher percentage of the immunotherapy group had rapid disease progression after initiating the therapy, compared with the chemotherapy group. As for the outcomes of HPD in NSCLC, all included studies revealed that HPD patients were associated with worse survival outcomes, compared with non-HPD patients. Because the accurate definition of HPD has not been well established, several studies have compared different definitions in their cohort. Kim CG reported that the concordance rate of HPD defined according to TGK (defined as the change in the sum of the longest diameters of the target lesions according to RECIST 1.1 criteria per month) and TGR was high (98.2%) [13]. However, Kim Y showed that HPD defined by the difference of total tumor volume is discordant with HPD defined by the difference of diameter of target lesions and the latter did not associate with worse OS [14]. A recent study, which included multiple cancer types, had reported that HPD measured by TGR was not associated with OS. Instead, HPD evaluated by RECIST had an impact on survival [18]. It remains to be clarified which definition of HPD would be better to separate this group of patients. Salvage chemotherapy was reported to be associated with improved overall response rates after PD-1/PD-L1 inhibitors [19–21]. A uniform definition of HPD would help to achieve early detection of HPD after ICIs and switch to chemotherapy for those still in good conditions.

Because HPD was significantly correlated with worse OS, it is important to identify biomarkers of HPD for patient selection before ICI treatment. Our study revealed that HPD had a significant correlation with ECOG, RMH, serum lactate dehydrogenase, the number of metastasis sites, and liver metastasis. Although several previous studies reported an association between HPD and other clinicopathological features, such as age > 65 years [4], female sex [22], neutrophil-to-lymphocyte ratio [14], and PD-L1 status [23], the present meta-analysis did not show a significant correlation. Higher serum lactate dehydrogenase reflected the intratumor hypoxia and was associated with worse survival outcomes [24]. Serum lactate dehydrogenase induced the upregulation of PD-L1 in lung cancer cells which might result in accelerated tumor growth [25]. Consistently, liver-induced immune tolerance to anti-PD-1/PD-L1 might explain the significant association between HPD and liver metastasis at baseline [26]. Low baseline ECOG PS was also correlated with a higher risk of HPD. Similarly, an association between resistance to immune checkpoint inhibitors and low baseline PS had been reported in NSCLC [27]. In addition to poorer RMH score, sporadic studies demonstrated that HPD might be associated with other prognostic scoring systems, such as the Gustave Roussy Immune score, lung immune prognostic index, and MD Anderson Cancer Center risk score, indicating that the selection of patients for ICI should be based on the prognostic score and general condition [13]. As the RMH score is also comprised of the number of metastatic sites (≤2 sites vs ≥3 sites) and elevated serum lactate dehydrogenase to predict patient survival, we assumed that HPD might have a close correlation with multisite tumor metastasis and elevated serum lactate dehydrogenase, which needs to be verified in further research. Many other clinicopathological features, such as platelet level in blood examination [17], metabolic tumor burden under positron emission tomography/computed tomography (PET/CT) [17], and MD Anderson Cancer Center risk score [15], have been reported to have a significant correlation with HPD. However, they have not been included in this meta-analysis owing to insufficient data.

Several studies have proposed different predictors to identify HPD. Kim CG identified lower frequencies of effector/memory subtypes (CCR7- CD45RA-) in CD8+ T cells and higher frequencies of severely exhausted cells (TIGIT+) in tumor-reactive PD-1+ CD8+ T cells to predict HPD, with the area under the curve reaching 0.926 and 0.938 [13]. This study emphasized the importance of pre-existing antitumor immune and the depth of T-cell exhaustion for selecting patients fit for immunotherapy. Zuazo-Ibarra found that the baseline of highly differentiated CD28 - CD27- CD4 T cells constituted a strong and reliable predictive biomarker for non-responders, including hyperprogressors, with 100% specificity and 75% sensitivity [28]. Tunali identified a clinical-radiomic model to predict HPD with the area under the curve reaching 0.865 [16]. Weiss demonstrated that quantitative chromosomal number instability score could provide a prediction accuracy of 92% for progression after immunotherapy [29]. Further studies are needed to explore the prediction accuracy of the chromosomal number instability score for HPD. The present meta-analysis identified 5 different clinical covariates that correlated with the odds of HPD which might also help select patients for ICI treatment.

The mechanism of HPD has not been well understood. Innate and adaptive immune systems might both play significant roles in the development of HPD. Lo Russo revealed that M2-like CD163^+^ CD33^+^ PD-L1^+^ tumor-associated macrophages can block anti-PD-1 antibody functional activity by interacting with the Fc domain of the antibody [15]. Increased T-regulatory cells in tumor-infiltrating lymphocytes have been reported to promote tumor progression after treatment with ICIs. Kamada found that PD-1 blockade may facilitate the proliferation of highly suppressive PD-1+ T-regulatory cells, resulting in the inhibition of antitumor immunity [30]. The upregulation of alternative immune checkpoints and cancer cell–intrinsic expression of PD-1 were proposed as potential mechanisms by which PD-1 blockade promoted tumor growth [31, 32]. Kim CG indicated that lack of pre-existing antitumor immune and T-cell exhaustion might promote the development of HPD after ICI [13]. Similarly, Zuazo-Ibarra found that HPD had a significant correlation with negative baseline highly differentiated CD4 T which reflected weaker potential anti-tumor capacities [28]. Further mechanism studies should be performed to elucidate the correlation between the baseline Immune environment and the development of HPD.

The present systematic review had limitations that should be considered when interpreting the results. First, this meta-analysis was based on published results rather than individual data, and hence the results remained inconclusive. In the assessment of the incidence and outcome of HPD, considering the existence of heterogeneity, further meta-analysis was not performed. Moreover, the inter-study variability of the definition of HPD might lead to heterogeneity among the included studies, and the current results should be interpreted with caution. Also, the number of studies included was limited, and some analysis only included three or four studies with a limited sample size. All of the studies included in this meta-analysis were retrospective. A control cohort was missing in 5 of 6 included studies. Further prospective randomized controlled trials were needed to clarify the results. Besides, some continuous variables that might correlate with HPD were not included in the present study because of insufficient data. Despite these limitations, this study provided a comprehensive understanding of HPD for further investigation.

## Conclusions

In conclusion, the present systematic review and meta-analysis summarized the clinical features of HPD in NSCLC after treatment with ICIs. Compared with patients with non-HPD, the OS of those with HPD was significantly worse. This meta-analysis indicated that Eastern Cooperative Oncology Group > 1, Royal Marsden Hospital score ≥ 2, serum lactate dehydrogenase > upper limit of normal, the number of metastasis sites > 2, and liver metastasis at baseline may correlate with the happening of HPD.

## Data Availability

The datasets used and/or analysed during the current study are available from the corresponding author on reasonable request.

## Abbreviations

CI: Confidence interval
HPD: Hyperprogressive disease
HR: Hazard ratio
ICI: Immune checkpoint inhibitor
NSCLC: Non-small cell lung cancer
OR: Odds ratio
OS: Overall survival
PD-1: Programmed death-1
PD- L1: Programmed death ligand-1
RMH: Royal Marsden Hospital
TGK: Tumor growth kinetics
TGR: Tumor growth rate
